# Clinical and Histopathological Characteristics of Cutaneous Lymphoid Hyperplasia: A Comparative Study According to Causative Factors

**DOI:** 10.3390/jcm9041217

**Published:** 2020-04-23

**Authors:** Myoung Eun Choi, Keon Hee Lee, Dong Jun Lim, Chong Hyun Won, Sung Eun Chang, Mi Woo Lee, Jee Ho Choi, Woo Jin Lee

**Affiliations:** Department of Dermatology, Asan Medical Center, University of Ulsan College of Medicine, Seoul 05505, Korea; mechoi316@naver.com (M.E.C.); dr.kenny0530@gmail.com (K.H.L.); lim9087@naver.com (D.J.L.); chwon98@chol.com (C.H.W.); changse2016@gmail.com (S.E.C.); miumiu@amc.seoul.kr (M.W.L.); jhchoy@amc.seoul.kr (J.H.C.)

**Keywords:** B lymphocyte, causative agents, cutaneous lymphoid hyperplasia, skin, T lymphocyte

## Abstract

Cutaneous lymphoid hyperplasia (CLH) is a heterogeneous type of reactive lymphocytic infiltration resembling cutaneous lymphoma clinically and histopathologically. Few studies describe the relationship between the causative agents and histopathological and immunohistochemical characteristics of CLH. We investigated the clinical and histopathological characteristics of 50 patients with cutaneous CLH and analyzed them according to causative factors and predominant cell types (B or T cells). We retrospectively reviewed medical records to identify causative agents, and histopathological and immunohistochemical features. The majority of infiltrating lymphocytes were T cells (60%). T cell-dominant CLH showed papuloplaque lesions, whereas B cell-dominant CLH lesions were nodular. The infiltration pattern differed between T and B cells. In terms of prognosis, B-cell-predominant lesions tended to respond better to treatment than T-cell-predominant lesions. Hair dyes tended to be associated with multiple CLH lesions in older patients. CLH lesions associated with drugs were located on the head and neck. Insect bites were likely to cause a solitary papular lesion. Histopathologically, infiltration depth was located more superficially than other causes and featured intense eosinophilic infiltration. Thus, our study demonstrated that CLH presents different clinicopathological features according to causative agents and predominant lymphocytic types.

## 1. Introduction

Cutaneous lymphoid hyperplasia (CLH) is a reactive heterogeneous infiltration of benign T or B cell lymphocytes in response to a variety of stimulations, which simulate cutaneous malignant lymphoma clinically and histopathologically [1]. A variety of etiologies have been described, including tattoo dyes; vaccinations; metals used in piercings; infectious agents such as *Borrelia burgdorferi,* herpes simplex, herpes zoster, and molluscum contagiosum viruses; and drugs [2,3,4,5,6,7,8]. Because of epidemiological variations in infectious agents and cultural differences, the stimuli of CLH seem to be different in each country.

Histopathological analysis is the most effective way to distinguish CLH from malignant lymphoma [9]. There have been attempts to categorize CLH according to predominant infiltrating cell types, etiologies, and both clinical and histological presentation, but no definitive classification system has been established for CLH [9,10,11]. There have been limited studies on the relationship between predominant lymphocytic cell type and clinicopathological features of CLH [9]. Moreover, studies on CLH in relation to causative agents have been restricted to case reports or small case series [1,4,12].

The clinical course of CLH varies from spontaneous regression to progression to malignant lymphoma [9,13,14]. Progression of CLH is rarely reported, and recurrences can occur after re-exposure to stimuli [13,14]. However, long-term follow-up data and differences in prognosis depending on the predominant cell type of CLH have not been evaluated. Our study investigated the clinical and histopathological characteristics of patients with CLH and analyzed them according to causative factors and dominant lymphocytic cell types.

## 2. Materials and Methods

### 2.1. Subjects

We identified a total of 50 patients with CLH who were evaluated at the Dermatology Department of the Asan Medical Center (Seoul, Korea) between January 2013 and December 2018. The medical records of these patients were reviewed to gather clinical, histological, and follow-up data. We only included cases in which both medical records and histopathological and immunohistochemical data were available. We excluded patients with other specific types of lymphoma and those who had a history of lymphoma.

### 2.2. Clinical Variables of Interest

The clinical features of the primary lesions, such as age at the time of diagnosis, sex, anatomical location of the lesion, morphology, multiplicity, clinical course, symptoms, time to diagnosis, and causative agents were identified from the medical records and clinical photographs. We defined drug-induced CLH if specific new drug was taken before the development of lesions and the discontinuation of the drug resulted in partial or complete resolution of lesions. We designated trauma-induced CLH only when the initial lesions were developed within the site where any kind of trauma was committed. Arthropod bite-related lesions were defined when the patient reported the insect bite and CLH initially developed at the exact location where the insect bite occurred. Cutaneous lymphoid hyperplasia associated with hair dye was defined when the patient reported of multiple usage of hair dyes before the development of lesions and when other causes were excluded. We also tried to find the symptoms and signs infections and previous history of tattoo.

### 2.3. Histopathology and Immunohistochemistry

Biopsies of all patients were available for histopathological and immunohistochemical study. The following features were assessed: infiltration pattern; infiltrate density and distribution; dominant lymphocytic cell types; lymphoid follicle formation; epidermal changes; and accompanying inflammatory cells, such as eosinophils, plasma cells, and histiocytes. The infiltration pattern was categorized as diffuse, band-like, nodular, or follicular. Diffuse patterns were defined by scattered cellular infiltration into the dermis without aggregation. Band-like patterns were identified by inflammatory cells located in the upper dermis forming a linear band. Nodular patterns were defined by the presence of multiple well-defined round aggregations of lymphocytes. Follicular patterns were defined by the presence of reactive follicular structures.

CLH lesions were divided into T cell- or B cell-predominant groups, according to the dominant lymphocytic cell types. In mixed type cases, we classified the lesions as either T or B cell according to the more prevalent lymphocytic cell type.

Infiltrating lymphocytic cells were evaluated for expression of the following markers: CD4, CD8, and CD20. The scoring for density of mixed inflammatory cells was assessed with semiquantitative parameters as 0 (no staining), 1 (<10% positive staining), 2 (10–50% positive staining), or 3 (>50% positive staining), and scoring of 3 was considered to be dense infiltration.

### 2.4. Statistical Analysis

All data were statistically analyzed using SPSS (version 18.0; SPSS, Inc., Chicago, IL). *p* Values less than 0.05 were considered statistically significant. Comparisons between subgroups of patients according to predominant cell types and causative agents were performed using a χ^2^ test or Fisher’s exact test for categorical variables and a *t* test for continuous variables. The Kruskal–Wallis test was used when more than 3 subgroups were present.

## 3. Results

### 3.1. Clinical Manifestations

The clinical features of the patients are presented in Table 1. The mean patient age was 53.2 years (age range, 18–82 y); 24 patients were men and 26 were women. Most patients presented with papules (n = 17) or nodules (n = 13), and the remaining patients had plaques (n = 11) or maculopatches (n = 9). More than half of the lesions were located on the head and neck (n = 29); others were on the trunk (n = 9) or extremities (n = 16). A single lesion was identified in about half of the patients (n = 28). Multiple lesions at the same anatomic site were found in 18 patients, whereas multiple lesions at different anatomic sites were identified in 4 patients. Symptoms were absent in 16 patients. Among 34 patients with symptoms, pruritus was predominant in 30 patients, tenderness was predominant in 4 patients, and pain was predominant in 2 patients. The duration of skin lesions before diagnosis ranged from 1 to 205 months (mean, 14 months).

### 3.2. Histopathological Features

The histopathological features of the patients are summarized in Table 2. Twenty-one patients (42%) had diffuse patterns, 21 patients (42%) had nodular patterns, 1 patient (2%) had a band-like pattern, and 7 patients (14%) had follicular infiltration. Among 7 patients with lymphoid follicle formation, 5 patients had germinal centers with the mantle zone forming well-defined structures. Twelve patients showed mild epidermal change, including 2 patients with epidermal flattening, 6 patients with focal spongiosis, and 4 patients with mild interface dermatitis.

Tumors were mostly composed of small lymphocytes and of variable amounts of histiocytes and plasma cells. Eosinophils were noted occasionally. Twelve patients (24%) had prominent eosinophil infiltration. Many histiocytes were observed in 7 patients (14%). Infiltration in the upper dermis was observed in 11 patients (22%), in the lower dermis in 2 patients (4%), and throughout the whole depth of the dermis in 37 patients (74%). Extension into subcutaneous tissue was noted in 9 patients (18%). Eight patients showed lobular infiltrations, whereas 1 patient had mixed lobular and septal pattern.

Polymerase chain reaction analysis of the immunoglobulin heavy chain (*IgH*) gene and T cell receptor gamma (*TCR-γ*) gene rearrangements were performed in 11 patients. One patient had positive results for *TCR-γ* gene rearrangements, and 1 patient had *IgH* gene rearrangements. After receiving a topical steroid injection, the patient with *TCR-γ* gene rearrangement showed complete remission and recurrence was not detected during 2 years of follow-up. In the patient with *IgH* rearrangement, kappa and lambda staining did not show monoclonality of infiltrating cells.

### 3.3. Clinical and Histopathological Analysis According to Predominant B or T Cells

**Figure 1:**
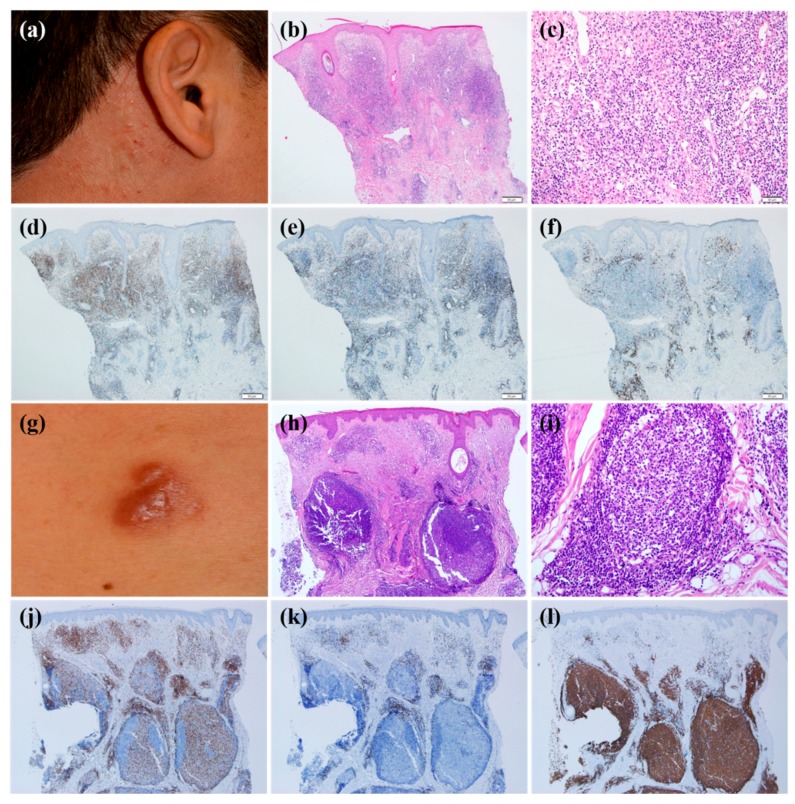
The clinicopathological features according to T cell- or B cell-dominant lymphocytic infiltration. (**a**–**f**) T cell-dominant cutaneous lymphoid hyperplasia. (**a**) Multiple erythematous papules developed in both periauricular areas. (**b**) Histopathological examination revealed diffuse pandermal lymphocytic infiltration (hematoxylin and eosin stain; magnification, ×40). (**c**) The cells were mostly composed of small lymphohistiocytes as well as small amounts of eosinophils (hematoxylin and eosin stain; magnification, ×200). Immunohistochemical staining revealed that most lymphocytes showed positivity with (**d**) CD4, whereas (**e**) CD8 and (**f**) CD20 were stained in relatively smaller proportions (×40). (**g**–**l**) B-cell-dominant cutaneous lymphoid hyperplasia. (**g**) Multiple erythematous nodules were noted in the chest area after trauma. (**h**) Skin biopsy analysis showed lymphoid follicular formation in the dermis: hematoxylin and eosin stain; original magnification, ×40; (**i**) hematoxylin and eosin stain; original magnification, ×200. Lymphocytes were weakly positive for (**j**) CD4 and (**k**) CD8 in the immunohistochemical staining, whereas most lymphocytes were densely stained with (**l**) CD20 (×40).

The immunohistochemical analyses showed that most infiltrating lymphocytes were B cells (40%) or T cells (60%). In T cell-dominant patients, CD4-positive T cells were more common than CD8-positive T cells. We analyzed the clinical and histopathological features according to dominant cell types (Table 3). The clinical morphology of CLH was statistically different depending on the dominant cell types (*p* = 0.001). T cell-dominant CLH lesions tend to present as papuloplaques (Figure 1a), whereas B cell-dominant CLH lesions were more prone to be nodules (Figure 1g) (*p* = 0.001). The mean age at the time of diagnosis, sex, location of lesion, multiplicity of lesion, presence of symptoms, and time to diagnosis were statistically insignificant. Histopathological analysis according to T cell- or B cell-dominance revealed that the infiltration pattern was different (*p* = 0.001). In other words, the T cell-dominant group had a diffuse pattern more frequently (Figure 1b–f) (*p* = 0.01), whereas the B cell-dominant group demonstrated a follicular pattern more often (Figure 1h–l) (*p* = 0.001). Infiltration density and depth, density of plasma cell, histiocyte, and eosinophil counts were not significantly different between the groups.

### 3.4. Clinical and Histopathological Analysis According to Causative Factors

Among 50 patients, 35 patients reported causative agents of cutaneous lesions. Seven patients reported using hair dye before the lesions developed, and 9 patients mentioned a previous history of an insect bite in the same area as the CLH lesion. Fourteen patients developed skin lesions associated with trauma, including 5 patients with injection site trauma and 3 patients with minor operation site trauma, 1 patient with piercing, 2 patients who underwent laser treatments, 2 patients with trauma related to shaving, and 1 patient with trauma related to a dental procedure. Five patients reported that CLH lesions developed after taking new drugs.

**Figure 2:**
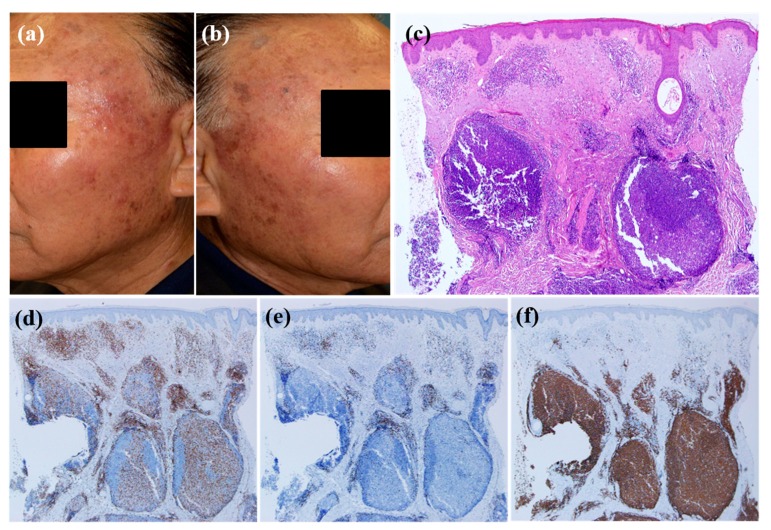
The clinicopathological characteristics of cutaneous lymphoid hyperplasia after hair dye use; (**a**,**b**) The patient had multiple erythematous nodules on his face after hair dye. (**c**) Skin biopsy reveals nodular and follicular infiltration in the dermis: Hematoxylin-eosin stain; magnification: ×40. Lymphocytes showed weak positivity for CD4 (**d**) and CD8 (**e**), while most lymphocytes stained positive with CD20 (**f**) (×40).

**Figure 3:**
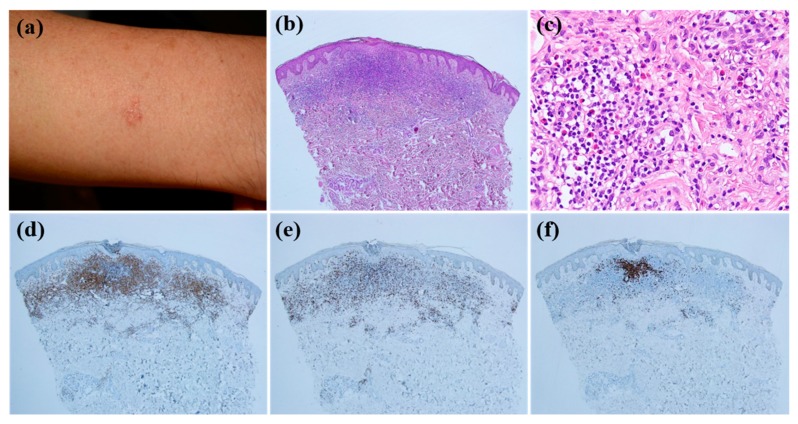
The clinicopathological characteristics of arthropod-bite reaction; (**a**) solitary erythematous papule was noticed on the patient’s forearm. (**b**) Histopathological examination reveals lymphocytic infiltration mostly on the upper dermis. (**c**) Numerous eosinophils were observed. (b: Hematoxylin-eosin stain; magnification: ×40 c: Hematoxylin-eosin stain; magnification: ×200). Lymphocytes showed positive stains for CD4 (**d**) and CD8 (**e**) and weak positive staining for CD20 (**f**) (×40).

**Figure 4:**
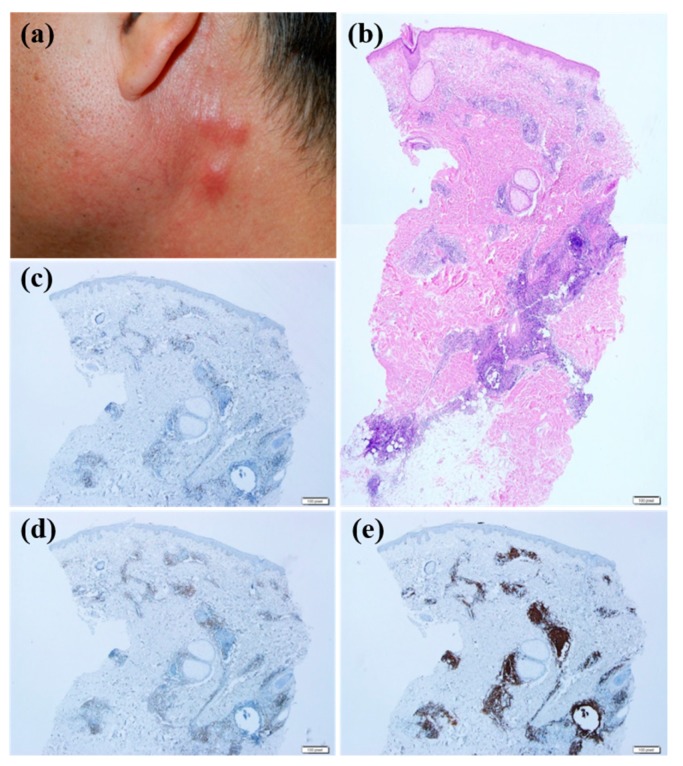
The clinicopathological features of trauma-associated cutaneous lymphoid hyperplasia; (**a**) multiple erythematous nodules were observed after injection-related trauma. (**b**) Biopsy specimen of the nodule reveals lymphocytic infiltration into deep dermis and subcutis (hematoxylin-eosin stain; magnification: ×20). Only small portions of lymphocytes showed positive reactivity for CD4 (**c**) and CD8 (**d**), while most lymphocytes showed positivity for CD20 (**e**) (×40).

**Figure 5:**
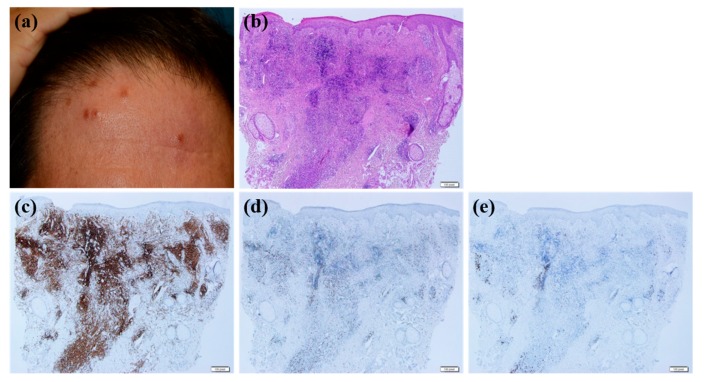
The clinicopathological features of drug-induced cutaneous lymphoid hyperplasia. (**a**) Multiple erythematous papules were observed 1 month after taking new drugs. (**b**) Histopathological examination revealed diffuse lymphocytic infiltration in the whole dermis (hematoxylin and eosin stain; magnification, ×40). Lymphocytes were intensely stained with CD4 (**c**), whereas fewer than 10% of lymphocytes showed positive reactivity for CD8 (**d**) and CD20 (**e**) (×40).

Patients whose skin lesions were related to hair dye tended to be older compared with those with other causative factors (mean age, 67.2) (*p* = 0.029). All patients with CLH related to hair dye use had multiple lesions (Figure 2a,b) except for 1 patient (*p* = 0.038). Six of 7 patients with CLH related to hair dye had lesions located on the face (Figure 2a,b) (*p* = 0.219), and the same number of patients had nodular or follicular infiltration (Figure 2c–f) (*p* = 0.180). CLH associated with insect bite history tended to be solitary (*p* = 0.028) and papular (Figure 3a) (*p* = 0.059). Five of 9 lesions were in the extremities (Figure 3a) (*p* = 0.124). Histopathologically, infiltration depth was located more superficially than that of other causes (Figure 3b–f) (*p* = 0.046). In other words, two-thirds of lesions caused by insect bites had dense lymphocytic infiltration on the upper dermis. Moreover, they showed intense eosinophilic infiltration, defined by a semiquantitative parameter of more than 2 (Figure 3c) (*p* = 0.027). However, in case of spider bites, infiltrations can also involve deeper dermis in contrast to other insect bites. When trauma is related to the formation of CLH, patients were relatively younger than those with CLH caused by other agents (mean age, 42.7; *p* = 0.060), and 78.6% of these patients had dense B cell infiltration at the site of the lesion (Figure 4a–e). Drug-related CLH lesions were all located on the head and neck area (Figure 5a) (*p* = 0.033). Four of 5 patients with drug-related lesions had intensive pandermal infiltration predominantly featuring CD4-positive T cells (Figure 5b–e).

### 3.5. Treatment and Follow-Up

Table 4 shows the summary of the treatment and follow-up for the 50 patients with CLH included in this study. The mean follow-up period was 14.0 months. Two patients did not receive treatment; of these, 1 patient refused the treatment and the other reported that his lesion regressed spontaneously. Thirteen patients were treated with only 1 modality, whereas 35 patients received multiple treatment types. The lesions were excised in 8 patients, none of whom experienced recurrence after excision. Nine patients were treated with oral corticosteroids, 3 patients with immunosuppressants such as methotrexate (n = 2) and cyclosporine (n = 1), 4 patients with hydroxychloroquine, 2 patients with dapsone, and 20 patients with antihistamines. Nineteen patients received local corticosteroid injections, 23 patients used topical corticosteroids, 8 patients used topical tacrolimus, and 1 patient was treated with radiotherapy.

During the follow-up period, the lesions of 16 patients completely resolved, 19 patients showed partial regression of their lesions, 7 patients maintained their lesions, 1 patient progressed to lymphoma, and 7 patients were lost to follow-up. Among the 16 patients whose lesions were completely resolved, 8 patients underwent surgical excision, 2 patients took steroids, and 6 patients were treated with topical or intralesional steroids. When classifying the patients into treatment-response groups (complete response, partial response, or no response) according to the dominant cell type, B cell-dominant lesions showed a significantly better response to treatment than T cell-dominant lesions (*p* < 0.001). However, the prognosis of patients according to causative agents did not differ significantly (*p* = 0.604).

## 4. Discussion

CLH is a lymphoid response that occurs in the presence of a variety of stimuli. A wide range of agents that are associated with the development of CLH have been identified and vary between countries, owing to differences in epidemiology, culture, and medical practice. Moreover, clinicopathological features have rarely been studied in relation to causative agents. In this retrospective study, we identified some relationships between causative agents and clinicohistopathological characteristics. We analyzed the patients by separating them into 4 groups based on causative agent: (1) trauma-related, (2) hair dye-associated, (3) arthropod bite reaction, and (4) drug-induced. The clinical and histopathological findings were closely related to the causative agents. Trauma-related CLH lesions mostly occurred on exposed sites such as the extremities and face. Hair dye-associated CLH typically presented with multiple papulonodules on the face in relatively old patients. Arthoropod bite reactions were associated with solitary lesions, and the infiltrations were dense but superficially located with numerous eosinophils. Lastly, drug-induced CLH lesions typically presented on the face and neck area. Although the findings were not statistically significant because of the small sample sizes, several interesting results were found. In trauma-related lesions, patients were relatively younger and 78.6% of patients showed dense B cell infiltration at the lesion site. On the other hand, 80% of patients revealed intensive pandermal infiltration, predominantly with CD4-positive T cells, in drug-related lesions. When reviewing previous case reports that analyzed clinicopathological features according to the causative agents mentioned above, some were comparable to our results but others showed somewhat different manifestations [1,15,16,17]. Moreover, a variety of pathological features can be manifested according to specific agents in the same group. For instance, insect bites generally show superficial lymphocytic infiltrations with eosinophils, and spider bites can reveal mixed inflammation to deeper dermis, large zones of necrosis, and signs of vasculitis [18]. Because of the small sample size and limited methods for investigating the causal relationships, further studies are needed to confirm our observed results. However, our results may help clinicians deduce the causative agents based on clinical and histopathological findings and enable them to help guide patients to avoid recurrence.

The male-to-female ratio of CLH was almost equal in our retrospective study, and the mean age of diagnosis was 53.2 years. This is similar to previous studies [9,10]. The most common location was the head and neck area, which was also similar to previous studies [10,19]. The head and neck area can be exposed to a variety of chemicals. In addition, cosmetic manipulation, including plastic surgery, cosmetic injection, and piercing, is common in these areas. Importantly, pruritus frequently accompanied the development and aggravation of CLH, which indicates a reactive pattern. When analyzing clinical features according to dominant cell types, T cell-dominant CLH lesions tended to present as papuloplaques, whereas B cell-dominant CLH lesions were prone to be nodules. Our results suggested that assessing the morphology of CLH lesions can imply histological features.

Moreover, we observed that infiltration patterns differed according to the dominant cell type. The T cell-dominant group frequently showed a diffuse pattern, whereas the B cell-dominant group had a follicular pattern more often. This finding may be associated with the morphological differences between T cell- or B cell-dominant CLH. It is likely that diffuse, top-heavy, or pandermal infiltration of T cells tends to be manifested as papuloplaques, whereas follicular infiltration of B cells is prone to present clinically as deeply situated nodules.

Although the general prognosis of CLH is good, clinicians may encounter resistant or progressive cases. Excluding patients lost to follow-up, 81% of patients showed some degree of improvement after treatment whereas 19% did not respond to treatment. One patient progressed to lymphoma despite receiving multiple modalities of treatment. Interestingly, B cell-dominant lesions showed a significantly better response to treatment than T cell-dominant lesions. Although various explanations can be given, B cell-dominant CLHs are associated more with trauma, whereas T cell-dominant CLHs are mostly caused by drugs or insect bites, which can act as more persistent stimuli without being noticed as causes. Because there have been limited reviews of the prognosis of CLHs based on dominant lymphocyte type, our observations are meaningful and useful for clinicians to predict treatment response based on biopsy results.

There are several limitations in our study. We could not find patients whose CLH lesions are related to causes that did not fit into any predetermined categories, such as infections and tattoos. Also, owing to the rarity of the disease, the small sample size and retrospective design limited more robust analysis.

## 5. Conclusions

In summary, we analyzed the clinical and histopathological features of 50 patients diagnosed with CLH. Despite the small sample size and retrospective study design, our study was able to analyze patients according to the dominant cell types and causative agents. Further studies are needed to classify this entity and to find more clues to correlate responsible agents.

## Figures and Tables

**Table 1:** Clinical features and causes of cutaneous lymphoid hyperplasia.

Clinical Parameters	Number of Patients (%)
Number of patients	50
Men	24
Women	26
Age range, y	18–82
Mean	53.2
**Lesion morphology**	
Papules	17 (34%)
Nodules	13 (26%)
Plaques	11 (22%)
Maculopatches	9 (18%)
**Site of presentation**	
Head and neck	29 (58%)
Trunk	9 (18%)
Upper extremities	10 (20%)
Lower extremities	6 (12%)
**Number of lesions**	
Solitary	28 (56%)
Multiple (at the same anatomic site)	18 (36%)
Multiple (at different anatomic sites)	4 (8%)
**Symptoms**	
Itching	30 (60%)
Pain	2 (4%)
Tenderness	4 (8%)
Asymptomatic	16 (30%)
**Cause**	
Unknown	15
Hair dye	7
Insect bite	9
Trauma	14
Drug	5
**Time to diagnosis, mo**	
Range	1–205
Average	14.0

**Table 2:** Histopathological features of cutaneous lymphoid hyperplasia.

Histopathological Parameters	Number of Patients (%)
Infiltration pattern	50
Diffuse	21 (42%)
Nodular	21 (42%)
Band-like	1 (2%)
Follicular	7 (14%)
Infiltration depth	50
Upper dermis	11 (22%)
Lower dermis	2 (4%)
Whole dermis	37 (74%)
Formation of lymphoid follicle	7
Well-defined structure	5 (71.4%)
Not well-defined structure	2 (28.6%)
Type of lymphocyte	50
B cell	20 (40%)
T cell	30 (60%)
**Molecular studies**	
IgH rearrangement	11
Positive result	1
Negative result	10
TCR- γ rearrangement	11
Positive result	1
Negative result	10

**Table 3:** Clinicohistopathological characteristics of cutaneous lymphoid hyperplasia patients according to dominant lymphocyte type (T cell vs B cell).

Clinical Features	T Cell	B Cell	*p* Value	Histopathological Features	T Cell	B Cell	*p* Value
**Sex**			0.580	**Infiltration pattern**			0.001
Men	15/30	10/20					
Women	15/30	10/20		Nodular	12/30	9/20	
**Age of diagnosis**	50.8	51.6	0.874	Diffuse	17/30	4/20	
**Causative agents**			0.269	Follicular	0/30	7/20	
Hair dye	3/20	4/15		Band-like	1/30	0/20	
Insect bite	7/20	2/15		**Infiltration density**			0.541
Trauma	6/20	8/15					
Drug	4/20	1/15		1	0/30	1/20	
**Location of the lesion**				2	16/30	9/20	
Face	16/30	13/20	0.413	3	14/30	10/20	
Trunk	7/30	2/20	0.285	**Infiltration location**			0.424
Upper extremity	4/30	6/20	0.171				
Lower extremity	5/30	1/20	0.381	Upper	7/30	4/20	
**Number of lesions**			0.580	Lower	0/30	1/20	
Multiple	17/30	10/20		Pandermal	23/30	15/20	
Single	13/30	10/20					
**Symptoms**			0.170	**Eosinophil counts**			0.740
Yes	19/30	17/20					
No	11/30	3/20		Score 0 or 1	22/30	16/20	
**Morphology of lesion**			0.001	Score 2 or 3	8/30	4/20	
Papuloplaque	23/50	5/20		**Plasma cell counts**			0.600
Maculopatch	4/30	5/20					
Nodular	3/30	10/20		Score 0 or 1	29/30	20/20	
**Time to diagnosis**	21.4	29.2	0.348	Score 2 or 3	1/30		
**Response to treatment**			0.08	**Histiocyte counts**			0.410
Complete response	9/27	7/15					
Partial response	11/27	8/15		Score 0 or 1	25/30	18/20	
No response	7/27	0/15	0.033	Score 2 or 3	5/30	2/20	

**Table 4:** Treatment modalities and clinical courses of cutaneous lymphoid hyperplasia.

Clinical Parameters	Number of Patients (%)
**Follow-up, mo**	
Range	1–95
Mean	14.0
**Treatment**	
None	2
Single	13
Multiple	35
**Therapy modalities**	
Surgical excision	8
Oral corticosteroids	9
Immunosuppressant	3
Hydroxychloroquine	4
Dapsone	2
Antihistamine	20
Local corticosteroids injection	19
Topical corticosteroids application	23
Topical tacrolimus application	8
Local radiotherapy	1
**Clinical course**	
Lost to follow-up	7
Complete resolution	16
Partial regression	19
Maintenance	7
Progression to lymphoma	1
